# Lymphocytic Choriomeningitis Virus Infection Demonstrates Higher Replicative Capacity and Decreased Antiviral Response in the First-Trimester Placenta

**DOI:** 10.1155/2019/7375217

**Published:** 2019-02-07

**Authors:** Elizabeth Ann L. Enninga, Regan N. Theiler

**Affiliations:** Department of Obstetrics and Gynecology, University of Texas Medical Branch, 301 University Blvd, Galveston, Texas, USA

## Abstract

Lymphocytic choriomeningitis virus (LCMV) is a rodent disease that can be transmitted to humans. A majority of persons infected with LCMV have only minor symptoms; however, it can cross the placental barrier during pregnancy and cause congenital defects in the fetus. Some viral infections early in gestation are hypothesized to lead to worse outcomes compared to those acquired during late gestation; however, LCMV has not been studied in this context. In the present study, differences in immunomodulation between the first- and third-trimester placental explants infected with LCMV were measured. LCMV replication was observed in the first-trimester chorionic villi, but not in term. The term placenta exhibited a robust innate immune response to infection by LCMV, marked by induction of *ifn*-*α*, *il*-*6*, and *tnf*-*α* gene expression which was not seen in the first-trimester explants. Cytokine secretion was also only seen in term explants. The results indicate that the first-trimester and term placentas differ in their permissiveness for LCMV infection, inversely correlating with the innate antiviral responses. This has implications for developing effective mechanisms that protect the fetus from infection based on stage of development.

## 1. Introduction

Lymphocytic choriomeningitis virus (LCMV) is an arenavirus native to rodents which is shed at high levels through excrement [1]. Although mice are the most common reservoir for LCMV, humans can acquire it by direct contact with fomites, through breathing in aerosolized virus or through organ transplantation [2]. Infection with LCMV as an adult or child is similar to symptoms of meningitis and will lead to a full recovery. However, if contacted during pregnancy, this single-stranded RNA virus can cause transplacental human fetal infections with serious clinical consequences [3]. Like many congenital pathogens, LCMV has a tropism for fetal neural and retinal tissue, causing issues with brain development including microencephaly, periventrictular calcification, cerebellar hypoplasia, and hydrocephalus [4, 5]. Meta-analysis demonstrated that children with congenital LCMV infection have a 35% mortality rate by approximately 2 years of age; those who survive have long-term neurological impairment and/or vision impairment [5]. The incidence of congenital LCMV is unknown, and infants with suspected congenital infection are not commonly tested for this viral pathogen. However, 9% of mice carry LCMV and 5% of humans are seropositive for the virus [6, 7], indicating that it may be an underdiagnosed etiology.

Congenital viral infections generally manifest with more severe fetal disease following the first-trimester maternal infection, when compared to infection later in gestation. For example, fetuses infected with Zika virus during the first trimester are known to be at increased risk for structural abnormalities [8, 9]. In a rat model of LCMV, pups introduced to the virus early in gestation (days 1-10) had more frequent and severe neuropathologies compared to pups exposed later in gestation [10]. In part, this effect has been attributed to the teratogenic impact of infection during early fetal development—most evident after early transplacental rubella and varicella infections [11, 12]. However, maternal-fetal immune interactions evolve throughout pregnancy [13], possibly changing the placental response to viral pathogens as pregnancy progresses.

Currently, no standard *in vitro* models exist for the study of the pathophysiology of human congenital viral infections. LCMV serves as a model virus for induction of robust innate and CD8^+^ T cell immune responses in the murine model and has been shown to activate innate immunity through Toll-like receptor- (TLR-) 2 [14, 15]. Human placental tissue explants, especially those from the first and third trimesters, are useful models for studying viral infection as well as development, toxicology, and cellular interactions [16]. Other investigators have studied infection of the first-trimester placental explants with human immunodeficiency virus (HIV) and human cytomegalovirus (CMV) which are known to transit the placenta [17, 18].

The present study is aimed at using human placental explants to model LCMV infection and study differences in the innate immune response during the first and third trimesters. The working hypothesis is that antiviral responses will be activated in placental tissues from term pregnancies, but not in those from early pregnancy. In addition, a robust immune response from placental tissue may suppress LCMV replication.

## 2. Materials and Methods

### 2.1. Viruses

This study utilized LCMV strain Armstrong RHK 11.7.1989 (Sealy Center for Vaccine Development, University of Texas Medical Branch), which was propagated on Vero E6 cells (ATCC). Titer was determined by plaque assay in Vero E6 cells with 0.5% agarose overlay, followed by neutral red staining on day 4 postinfection.

### 2.2. Human Placental Explants

This study was approved by the University of Texas Medical Branch (UTMB) Hospital Institutional Review Board. Tissue was collected through a deidentified biobank, providing only the gestational age at collection and confirmed live singleton pregnancy. Eligible patients were enrolled in the study at the time of term cesarean delivery (≥37 weeks) or elective termination of pregnancy (5-14 weeks). Patients were at least 18 years of age and were excluded from the study if they had fever, preterm labor, HIV, syphilis, hepatitis B or C, or other clinically evident infections. Tissues were handed off aseptically, and chorionic villi were dissected from beneath the chorionic plate and washed in phosphate-buffered saline (PBS). They were cut into explants approximately 1-3 mm in diameter and suspended in 75 *μ*m Netwells (Corning) containing RPMI 1640 with 1 mM HEPES, penicillin, streptomycin, amphotericin B, and 10% FBS. Explants were maintained at 37°C with 8% O_2_ as previously described [16]. Explants were inoculated with 2 × 10^4^ pfu/mL LCMV (1 : 1000 stock dilution) for 2 hours before the media was changed. The media was sampled daily for viral titer. Explants for tissue harvest were washed in cold PBS and lysed on ice by sonication, and supernatant was collected by centrifugation to remove cellular debris. Supernatant was passed through a 200 *μ*m filter prior to dilution in RPMI 1640 for plaque assay.

### 2.3. RT-PCR

200 mg explant tissue was incubated with 12 *μ*g/mL polyI:C with FuGENE transfection agent (Promega, Madison, Wisconsin) or 2 × 10^4^ pfu/mL LCMV for 24 hours and transferred into RNAlater (Thermo Fisher Scientific, Waltham, Massachusetts). Total tissue RNA was extracted using RNAqueous kit (Thermo Fisher Scientific) according to the manufacturer's instructions. Real-time amplification was carried out using the Applied Biosystems 7500 Fast System with TaqMan® reagents according to the manufacturer's instructions (Thermo Fisher Scientific). TaqMan® gene expression assays were used with probes for *ifn*-*γ* (Hs0017443_m1), *ifn*-*α* (Hs00356648_s1), *il*-*6* (Hs00174131_m1), *tnf*-*α* (Hs00174128_m1), *il*-*29* (Hs00601677_g1), and *il*-*28* (Hs00820125_g1). Expression was compared to that of *β*-actin, and four seperate subjects were used for each experiment run in triplicate.

### 2.4. Protein Determination

Placental explants with 12 *μ*g/mL polyI:C with FuGENE or 2 × 10^4^ pfu/mL LCMV were incubated for 24 h, and media was collected to determine baseline secretion of interferon- (IFN-) *α*, interleukin- (IL-) 2, IL-6, IFN-*γ*, and tumor necrosis factor- (TNF-) *α*. Concentrations of proteins were measured by Bio-Plex assay according to the manufacturer's instructions (Bio-Rad, Hercules, California). Lactate dehydrogenase (LDH) cytotoxicity assays were used to quantify cell death (Thermo Fisher Scientific). Four separate placentas from the first trimester and term were analyzed in duplicate.

### 2.5. Statistical Analysis

ELISA and RT-PCR data are expressed as mean ± standard deviation. Comparisons between the first and third trimesters were completed by a nonparametric *t*-test. For multiple comparison analysis, one-way ANOVA with the Bonferroni-Sidak correction was utilized. Significance was defined as a *p* value ≤ 0.05.

## 3. Results

### 3.1. LCMV Does Not Replicate in the Term Placenta

**Figure 1:**
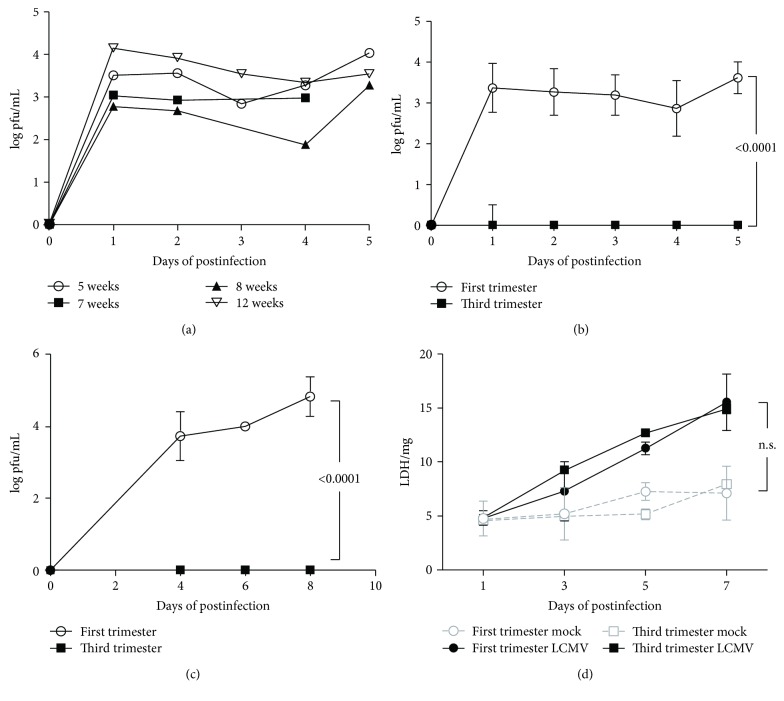
LCMV replication is more robust in the first-trimester placental explants. (a) The first-trimester human placental explants are permissive to viral replication at multiple gestational ages (*n* = 1/group). (b) LCMV titer placental villus explant tissue lysates from the first trimester and term. (c) LCMV viral secretion into the media starting at day 4 after infection. (d) LDH release by the first-trimester and term placentas. Data is presented as the mean and standard deviation of four different subjects in each group. Significance (*p* ≤ 0.05) was determined by ANOVA.

First, placental explants were infected with 2 × 10^4^ pfu/mL LCMV, and then media was collected for up to 5 days to measure viral secretion.  Figure 1(a) demonstrates that explants from 5-12 weeks of gestation actively secreted the virus; however, term (third trimester) placental explants did not support replication of LCMV (Figure 1(b)). Additionally, tissue lysates demonstrated viral replication in the first trimester tissue but not in the third trimester in 4-8 days of postinfection (Figure 1(c)). The lack of replication in the term placenta versus the first trimester was not due to differences in cell viability as measured by LDH secretion (Figure 1(d)). Although a trend was visible, no difference in LDH secretion was observed between the first and third trimesters and infected versus uninfected explants (*p* = 0.1515).

### 3.2. LCMV Induces Antiviral Responses in the Term, but Not in the First-Trimester, Placenta

**Figure 2:**
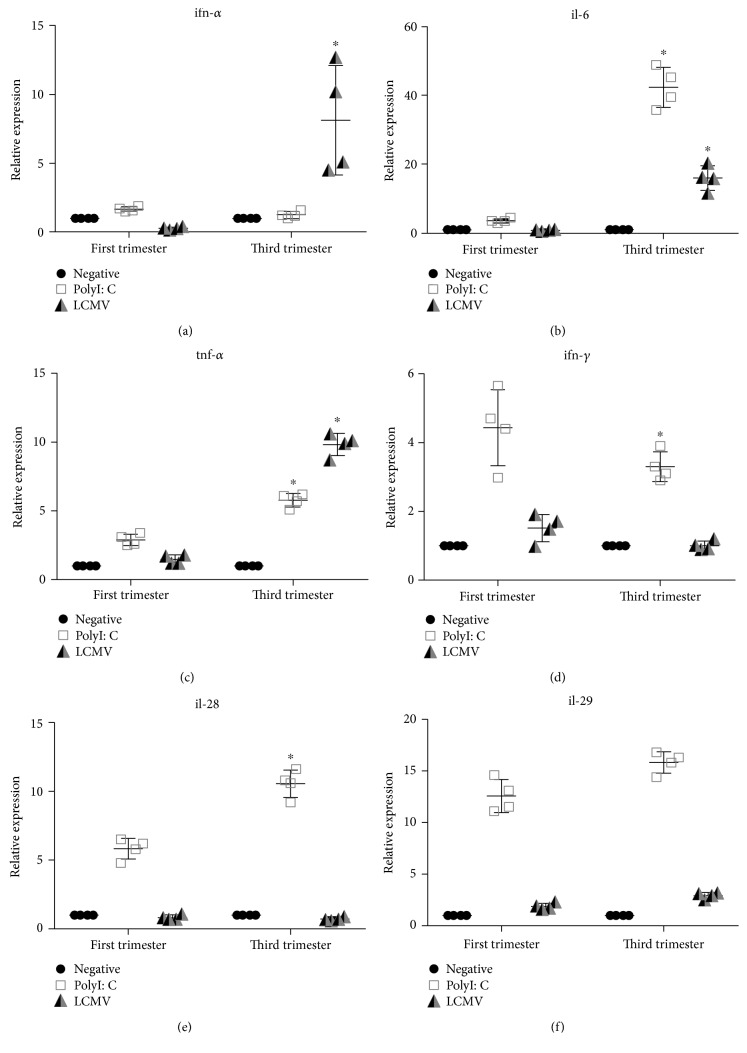
Innate immune responses are activated in term, but not in the first-trimester, human placental explants infected with LCMV. Term and the first-trimester placental explants were analyzed for mRNA expression by RT-PCR. (a) *ifn*-*α*, (b) *il*-*6*, (c) *tnf*-*α*, (d) *ifn*-*γ*, (e) *il*-*28*, and (f) *il*-*29*. Graph represents relative quantities and standard deviation of mRNA normalized to *β*-actin. Significance is defined as a *p* value ≤ 0.05 comparing the first vs. third trimester, denoted by ^∗^ (*n* = 4 per group).

Using real-time, quantitative RT-PCR, we analyzed induction of antiviral responses in the term and first-trimester placenta after treatment with polyI:C (synthetic RNA analog that signals through TLR-3) or LCMV for 24 hours. Each experiment was performed using tissue from four different subjects. Graphs represent the evaluation of differences between trimesters, analyzed by ANOVA (Table 1). Basal cytokine mRNA levels were not altered between the first-trimester and third-trimester tissues. LCMV induced mRNA expression of *ifn*-*α* (Figure 2(a)), *il*-6 (Figure 2(b)), and *tnf*-*α* (Figure 2(c)) in the term, but not the first-trimester, placenta. In response to TLR agonist polyI:C, *il*-6 and *tnf*-*α* had a more robust mRNA production following stimulation in the term placenta than in the first-trimester explants. Interestingly, *ifn*-*γ* followed an opposite pattern, trending toward lower transcript production in the third-trimester compared to the first-trimester tissue (Figure 2(d)). Expression of type III interferons *il*-*28* and *il*-*29* was also examined in LCMV infection. No increase in mRNA expression was demonstrated after LCMV infection in the first- or third-trimester tissue (Figures 2(e) and 2(f)). However, polyI:C did activate *il*-*28* transcription more robustly in the third-trimester explants compared to the first.

### 3.3. The First-Trimester Explants Do Not Secrete Cytokines following LCMV Infection

**Figure 3:**
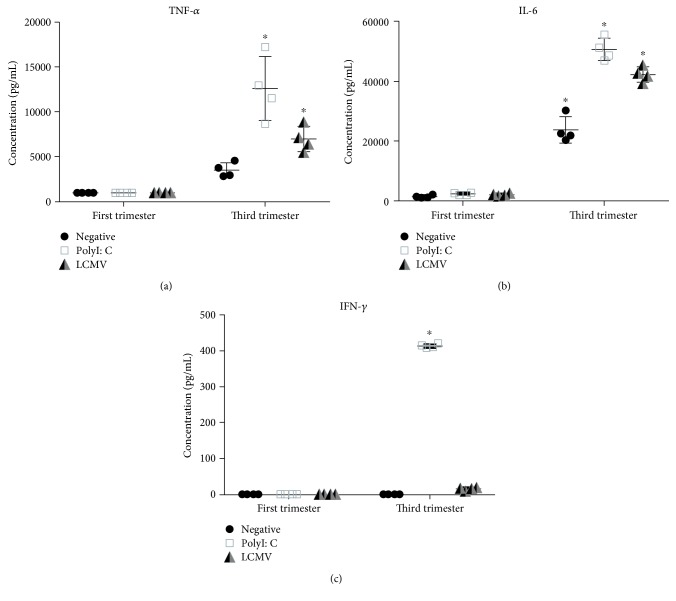
Cytokine secretion is seen in response to LCMV in term placental explants but not in the first-trimester tissue. Term and the first-trimester placental explants were analyzed for cytokine secretion after infection. (a) TNF-*α*, (b) IL-6, and (c) IFN-*γ*. Mean and standard deviation are shown. Significance is defined as a *p* value ≤ 0.05 comparing the first vs. third trimester, denoted by ^∗^ (*n* = 4 per group).

Bio-Plex assays were utilized to measure cytokine secretion of IFN-*α*, interleukin- (IL-) 6, IFN-*γ*, and TNF-*α* following infection with LCMV or stimulation with polyI:C. Each sample was run in duplicate, using the placentas from four individual subjects. Again, the graphs represent the differences in comparisons between trimesters. IFN-*α* concentration was under the limit of detection for this assay in both term and the first-trimester explant cultures. Increased TNF-*α* and IL-6 secretion was observed with the third-trimester placenta after LCMV or polyI:C incubation (Figures 3(a) and 3(b)). Conversely, this cytokine response was not seen in the placentas from the first trimester. PolyI:C induced a strong IFN-*γ* response in the term placenta but not in the first-trimester placenta (Figure 3(c)). This indicates that the antiviral immune response to LCMV and polyI:C was more robust in the term placenta than in the first-trimester tissue.

## 4. Discussion

Congenital viral infections can lead to fetal complications that can negatively affect the health of the baby, including birth defects or death. In addition, pregnant women infected by viruses, including varicella, measles, or flu, are at greater risk of complications and even death compared to nonpregnant women [19, 20]. Clearly, pregnancy impacts the maternal immune response, but mechanisms for increased maternal and fetal susceptibility to certain pathogens are unclear. Congenital viral pathogens can cross the fetal-maternal interface and infect trophoblast cells, Hofbauer cells, chorionic villi, and amniotic fluid, which have been extensively reviewed [21]. Lymphocytic choriomeningitis virus (LCMV), although less well characterized, is no exception. Though the incidence of LCMV infection during pregnancy is unknown, it has been shown to cause microcephaly, periventricular calcification, cerebellar hypoplasia, and hydrocephalus due to its tropism for neural tissue [3–5]. Data presented here characterize differences in LCMV permissiveness and antiviral responses in human explants from the first-trimester and term placenta.

First, LCMV was determined to replicate effectively and be secreted into the media of the first-trimester explants, regardless of gestational week. However, viral replication was not detected in tissue or media from term explants, and this effect was not due to increased cell death. It is possible that the term placenta may have developed protective mechanisms to deal with LCMV infection compared to the first trimester. This result corresponds with data from other viral infections that indicate early infection leads to worse fetal outcomes [8–10], adding new data that demonstrates LCMV infection is no exception to previous findings.

During LCMV infection, TLR2 and MyD88 knockout mice have shown decreased cytokine production and cytotoxic T cell responses resulting in persistent infection [15]. TLR2 expression in the human placenta is localized mainly to endothelial cells and macrophages; however, expression of TLR2 on syncytiotrophoblasts and fibroblasts is also seen [22]. Infection in the murine placenta also increases the expression of TLR2 as a protective mechanism [23]. PolyI:C is a well-defined double-stranded RNA analog that signals through intracellular TLR3, leading to expression of type I interferons (IFN-*α*/*β*), TNF-*α* and IL-6. The data demonstrate an increase in mRNA expression of innate cytokines in the third-trimester explants compared to the first-trimester explants exposed to both LCMV and polyI:C. Relative gene expression of *ifn*-*α*, *il*-*6*, and *tnf*-*α* in the term placentas infected with LCMV was higher compared to the first-trimester gene expression and followed a similar pattern to polyI:C stimulation. mRNA expression of type II interferon *ifn*-*γ* and type III interferons, *il*-*28* and *il*-*29*, was increased with polyI:C stimulation but not with LCMV infection, suggesting that these may not be critical regulators of innate responses to this virus. In the human placenta, IFN-*γ* expression decreases as pregnancy progresses, whereas the IFN-*γ* receptors are expressed throughout [24, 25], indicating that antiviral actions of this cytokine by trophoblasts are more likely to be crucial early in gestation but can respond to the release of IFN-*γ* by other immune cells, such as natural killer cells.

Interestingly, no protein production of IFN-*α* was observed, although TNF-*α* and IL-6 cytokine secretion was noted in term explant cultures, but not in the first-trimester cultures. Type I interferon responses are important modulators of viral infection in the placenta, and viruses have found ways to evade this response. In IFN-*α*/*β* receptor (IFNAR) knockout animals, viremia and death occurred in the dams and fetuses during pregnancy; however, transfer of embryos with functional IFNAR rescued these animals from demise [26]. Type I responses have also been shown to cause harm. IFNAR^+/−^ fetuses, but not IFNAR^−/−^, infected with Zika virus were reabsorbed due to abnormal development of placental vasculature, cellular apoptosis, and hypoxia initiated by IFNAR signaling [27, 28]. Therefore, there is a critical balance between protective and harmful inflammations through type I interferons in placental viral infection.

Limitations of the current study include the use of tissue explants, which do not discern which cell types are infected by LCMV and the small sample size used for each assay. Additionally, the experiments do not detail whether infection occurs at the apical or basal side of infected cells since the LCMV receptor is known to change its location throughout placental development [29]. At this point, we can only hypothesize that interactions between LCMV and TLRs lead to NF-*κ*B or MyD88 activation and the release of TNF-*α* or IFN-*α*. Future experiments are needed to clarify the mechanism of cytokine induction by LCMV as well as the mechanism(s) of suppression of this response in the first-trimester placenta.

## 5. Conclusions

Together, the data do suggest that innate immune response to LCMV infection of the human placenta is more vigorous in the third trimester than in the first trimester. The absence of viral replication in term placental explants may be attributable to the robust innate antiviral response in this tissue. Such findings parallel the clinical observation of decreased transplacental transmission and less severe fetal phenotypes of viral pathogens acquired in later gestation. Continued research into LCMV as a model of human congenital infection and immunity is warranted.

## Figures and Tables

**Table 1:** ANOVA comparisons between the first-trimester and third-trimester explants.

Comparison	Mean (relative expression or pg/mL)	*p* value
*ifn*-*α*		
Neg.	1 vs. 1	0.999
PolyI:C	1.7 vs. 1.2	0.9777
LCMV	0.3 vs. 8.1	*0.0002*
*ifn*-*γ*		
Neg.	1 vs. 1	0.999
PolyI:C	4.4 vs. 3.3	*0.0178*
LCMV	1.5 vs. 1	0.4358
*il*-*6*		
Neg.	1 vs. 1	0.999
PolyI:C	3.6 vs. 42.4	*<0.0001*
LCMV	0.8 vs. 16	*<0.0001*
*tnf*-*α*		
Neg.	1 vs. 1	0.999
PolyI:C	2.9 vs. 5.8	*<0.0001*
LCMV	1.5 vs. 9.8	*<0.0001*
*il*-*28*		
Neg.	1 vs. 1	0.999
PolyI:C	5.8 vs. 10.6	*<0.0001*
LCMV	0.8 vs. 0.7	0.999
*il*-*29*		
Neg.	1 vs. 1	0.999
PolyI:C	12.6 vs. 15.8	*<0.0001*
LCMV	1.9 vs. 2.9	0.2201
TNF-*α* protein		
Neg.	1000 vs. 3536	0.11
PolyI:C	1000 vs. 12,604	*<0.0001*
LCMV	1000 vs. 6983	0.0002
IL-6 protein		
Neg.	1449 vs. 23,737	*<0.0001*
PolyI:C	2386 vs. 50,633	*<0.0001*
LCMV	2043 vs. 42,250	*<0.0001*
IFN-*γ* protein		
Neg.	0 vs. 0	0.9999
PolyI:C	0 vs. 414	*<0.0001*
LCMV	0 vs. 15.3	*<0.0001*

## Data Availability

The raw data used to support the findings of this study are available from the corresponding author upon request.
